# METTL3-mediated *N*^6^-methyladenosine modification governs pericyte dysfunction during diabetes-induced retinal vascular complication

**DOI:** 10.7150/thno.63441

**Published:** 2022-01-01

**Authors:** Long Suo, Chang Liu, Qiu-Yang Zhang, Mu-Di Yao, Yan Ma, Jin Yao, Qin Jiang, Biao Yan

**Affiliations:** 1 Eye Institute, Eye & ENT Hospital, Shanghai Medical College, Fudan University, Shanghai, China.; 2 The Affiliated Eye Hospital, Nanjing Medical University, Nanjing, China.; 3 The Fourth School of Clinical Medicine, Nanjing Medical University, Nanjing, China.; 4 NHC Key Laboratory of Myopia (Fudan University), Key Laboratory of Myopia, Chinese Academy of Medical Sciences, and Shanghai Key Laboratory of Visual Impairment and Restoration (Fudan University), Shanghai, China.

**Keywords:** Microvascular complication, Diabetic retinopathy, m^6^A methylation, Pericyte dysfunction

## Abstract

**Rationale:** Microvascular complication is a major cause of morbidity and mortality among the patients with diabetes. Pericyte dysfunction is the predominant pathological manifestation of microvascular complication. *N*^6^-methyladenosine (m^6^A) serves as the most prevalent modification in eukaryotic mRNAs. However, the role of m^6^A RNA modification in pericyte dysfunction is still unclear.

**Methods:** Quantitative polymerase chain reactions and western blots were conducted to detect the change of m^6^A RNA modification in pericytes and mouse retinas following diabetic stress. MTT assay, transwell migration assay, caspase 3/7 activity assay, calcein-AM/propidium iodide (PI) staining, and TUNEL staining were conducted to determine the role of METTL3 in pericyte biology *in vitro*. Retinal trypsin digestion, vascular permeability assay, and IB4-NG2 double immunofluorescent staining were conducted to determine the role of METTL3 in retinal pericyte dysfunction and vascular complication. RNA sequencing, RNA pull-down assays and immunoblots were conducted to clarify the mechanism of METTL3-mediated pericyte dysfunction and vascular complication.

**Results:** The levels of m^6^A RNA methylation were significantly up-regulated in pericytes and mouse retinas following diabetic stress, which were caused by increased expression of METTL3. METTL3 regulated the viability, proliferation, and differentiation of pericytes *in vitro*. Specific depletion of METTL3 in pericytes suppressed diabetes-induced pericyte dysfunction and vascular complication *in vivo*. METTL3 overexpression impaired pericyte function by repressing PKC-η, FAT4, and PDGFRA expression, which was mediated by YTHDF2-dependent mRNA decay.

**Conclusion:** METTL3-mediated m^6^A methylation epigenetically regulates diabetes-induced pericyte dysfunction. METTL3-YTHDF2-PKC-η/FAT4/PDGFRA signaling axis could be therapeutically targeted for treating microvascular complications.

## Introduction

Pericytes are recognized as the vascular mural cells embedded within the basement membrane of capillaries 1. They wrap around the endothelial cells (ECs) of capillaries and regulate vascular tone, cell-cell contact, and functional integrity of capillary units 2. Pericyte dysfunction is an initiator of microvascular remodeling, leading to endothelial dysfunction and microvascular complications in several tissues/organs such as kidney, skeletal muscle, brain, heart, and retina 3-5. Thus, modulating pericyte biology contributes to the improvement of clinical outcomes for microvascular diseases.

The retina provides a unique window that allows direct and non-invasive visualization of systemic circulation. Architectural alteration in retinal capillaries may induce microcirculatory dysfunction, which predisposes the occurrence of ocular diseases 6, 7. Diabetic retinopathy (DR) is a microvascular complication and is a leading cause of visual disability and blindness in the people with diabetes 8. The pathogenesis of DR is tightly associated with retinal capillary remodelling, progressive fibrosis, and retinal detachment. Hyperglycaemia causes pericyte dysfunction and retinal vascular complications, such as microaneurysm formation, blood-retinal barrier (BRB) breakdown, pericyte-EC interaction disruption, and pathological neovascularization 9-11. Several factors are contemplated to be engaged in pericyte dysfunction, such as oxidative stress, free radical, advanced glycosylation end product, and inflammatory stimuli 12-14. However, the mechanism by which diabetes persuades pericyte dysfunction is still unclear. Exploring the potential strategy for targeting pericyte dysfunction may provide novel insights for the treatment of microvascular diseases.

RNA modifications are ubiquitously present in almost all mRNAs 15. The structural diversity of modified RNAs provides the regulatory potentials for the organized metabolism and functions 16. *N*^6^-Methyladenosine (m^6^A) is the most prevalent chemical modification on eukaryotic mRNAs. Dynamic m^6^A mRNA modification is deposited by m^6^A methyltransferases, removed by m^6^A demethylases, and recognized by the reader proteins 17. m^6^A modification plays important roles in protein translation, RNA metabolism, and RNA splicing 15, 18. Dysregulation of m^6^A modification has been implicated in several human disorders, such as cancers, neurological diseases, and cardiovascular diseases 19. Nonetheless, the role of m^6^A RNA modification in pericyte dysfunction is still unclear.

In this study, we revealed that m^6^A RNA modification was dynamically regulated following diabetic stress and involved in the regulation of gene expression in pericytes. METTL3 regulated pericyte viability, proliferation, and differentiation *in vitro*. Pericyte-specific METTL3 knockout suppressed retinal pericyte dysfunction and vascular complication *in vivo*.

## Materials and Methods

### Animal experiment

All experiments were conducted according to the guidelines of the Association for Research in Vision and Ophthalmology (ARVO) Statement for the Use of Animals in Ophthalmic and Vision Research and approved by the Institutional Animal Care and Use Committee of the authors' institute. C57BL/6J (wild-type, WT) mice were purchased from the Nanjing Qinglongshan Experimental Animal Center (Nanjing, China). *Mettl3* floxed mice were purchased from GemPharmatech Co. Ltd (Nanjing, China). *Pdgfrβ-*Cre mice were purchased from Beijing Biocytogen Co. Ltd (Beijing, China) generated on C57BL/6J background. *Mettl3* ^flox/flox^ mice were crossed with* Pdgfrβ-*Cre mice to generate pericyte-specific *Mettl3* knockout mice. All mice were bred under the specific-pathogen free condition with free access to diet and water or their nursing mothers with alternating 12/12 light-dark cycle (lights on at 08:00 and off at 20:00).

### Cell culture and transfection

Human retinal pericytes (ACBRI-183) was obtained from Cell Systems Corp. (CSC, USA). Pericytes (Passages 5-8) were cultured in Dulbecco's modified Eagle's medium (DMEM) containing 5.5 mmol/L D-glucose supplemented with 10% fetal bovine serum (FBS, 10099141C, Gibco, USA) and 1% antibiotic-antimycotic solution (penicillin/streptomycin, 15140122, Gibco, USA). They were cultured at 37 °C in 5% CO_2_ and 95% humidity. Cell transfection was conducted at about 80% confluence using Lipofectamine 6000 (C0526, Beyotime, China) according to the manufacture's protocols.

### STZ-induced diabetic retinopathy

The mice (8-week old, male) were intraperitoneally injected with STZ in sodium citrate buffer (pH 4.5) for 5 consecutive days (50 mg/kg/day). The control groups were intraperitoneally injected with an equivalent volume of citrate buffer. They were allowed to access to the standard laboratory chow and water. Blood glucose levels were measured after STZ injection using the OneTouch Ultra meter (LifeScan, USA) one week after the final injection and then monitored biweekly. The mice with blood glucose levels ≥16.7 mM (300 mg/dL) were considered diabetic and included in the study as diabetic group 20.

### NG2/IB4 immunofluorescence staining

The retinas were isolated and fixed in cold 4% PFA for 15 min, washed in PBS for 5-10 min, and blocked in 1% TritonX-100/5% BSA for 30 min at 37 °C. The whole-mount retinas were stained with NG2 antibody (1:100, ab50009, Abcam, USA) overnight at 4 °C. The primary antibody was removed and 1 × PBST (0.1% Tween-20 in 1×PBS, pH 7.4) was used to wash the retinas 4 × 10 min. The retinas were then stained with the Alexa Fluor 594 goat anti-mouse IgG (1:500, A11005, Invitrogen, USA) for 3 h at room temperature to label pericytes. Endothelial cells were labeled by staining Isolectin B4 (IB4, 1:100, L2895, Sigma, USA) for 2 h at room temperature. The staining signaling was captured using a fluorescent microscope (IX73P1F, Olympus, Japan).

### Retinal trypsin digestion assay

Retinal vessels were isolated by trypsin digest method. Briefly, the mouse eyes were enucleated and fixed in 10% neutral formaldehyde for 24 h. They were harvested and gently shaken in water at room temperature overnight, and then incubated with 3% trypsin (1:250, 215250, BD Difco, USA) in 0.1 M Tris buffer (pH 7.8) at 37°C for 1 h when the tissue became loose. After trypsin digestion, the retinas received repeated washed in water. The network of retinal vessels were isolated and mounted on the glass slides. Dried retinal vessels were stained with the Glycogen Periodic Acid Schiff (PAS/Hematoxylin) Stain Kit (G1281, Solarbio, China) and observed under a light microscopy.

### Quantification of m^6^A RNA modification level

The levels of m^6^A mRNA modification were determined by the EpiQuik m^6^A RNA Methylation Quantification Kit (P-9005-48, EpiQuik, USA) according to the manufacturer's protocols. Pericytes were incubated with 25 mM glucose (High glucose, HG) for 24 h and 48 h. Retinal vessels were isolated out at 1-mont, 2-mont, 4-month, and 6-month after the induction of diabetes. Total RNAs were isolated from pericytes or retinal tissues using the TRIzol reagent. mRNAs were enriched by the Dynabeads mRNA Purification Kit (61006, Ambion, USA). Total RNAs were bound to strip wells using the RNA high binding solution. 200 ng poly-A-purified RNAs were used for each sample analysis. 200 ng RNAs were firstly coated on the assay wells. The suitable diluted concentration of capture antibody solution and detection antibody solution were then added to the assay wells, respectively. m^6^A levels were quantified by reading the absorbance at a wavelength of 450 nm of each well. The level of m^6^A RNA modification was proportional to the OD intensity measured. A standard curve was plotted to reflect the relative level of m^6^A RNA modification.

### m^6^A dot blot assay

Total RNAs were harvested from pericytes or retinal vessels using the Trizol reagent. mRNAs were enriched using the Dynabeads mRNA Direct Purification Kit. RNA quantity was monitored by the NanoDrop ND-1000 Spectrophotometer (Agilent, USA). mRNAs were denatured by heating at 95°C for 5 min and then chilled on the ice. Then, mRNAs were blotted onto Hybond N^+^ membranes (FFN13, Beyotime, China). UV crosslinking of RNAs to the membranes were conducted in an Ultraviolet Crosslinker. The membranes were blocked with 5% defatted milk for 1 h at room temperature. The membranes were incubated with m^6^A-specific antibody (1:1000, 202003, Synaptic Systems) at 4°C overnight. After three washes with 1 × PBST, the membranes were incubated in HRP-conjugated goat anti-rabbit IgG secondary antibody (1:2500, sc-2030, Santa Cruz) for 1 h at room temperature with gentle shaking. Finally, the membranes were washed again three times in 1 × PBST. The signals were detected by the SuperSignal West Dura Extended Duration Substrate (34075, Thermo Fisher Scientific). Quantified m^6^A levels were normalized to the amount of the loaded mRNAs.

### Methylated RNA immunoprecipitation-PCR (MeRIP-qPCR) analysis

Total RNAs were fragmented using the RNA Fragmentation reagent (AM8740, Invitrogen,) at 70 °C for 20 min. A small amount of fragmented RNAs was left aside as the input RNAs. The fragmented RNAs were immunoprecipitated using the m^6^A polyclonal antibody coupled to Dynabeads (10002D, Invitrogen) in MeRIP buffer (150 mM NaCl, 10 mM Tris-HCl, pH 7.5, 0.1% NP-40) for 2 h at 4 °C. m^6^A containing mRNAs were eluted twice with 6.7 mM N6-methyladenosine 5ʹ-monophosphate sodium salt (M2780, Sigma-Aldrich) at 4 °C for 1 h, precipitated with 5 mg glycogen (AM9510, Life Technologies), extracted with Acid Phenol (pH 4.3-4.7), and precipitated by ethanol. Immunoprecipitated RNAs were purified and reversely transcribed with the Oligo (dT) primers using the SMARTScribe Reverse Transcriptase (639537, Clontech, USA). Relative gene expression was determined by qRT-PCR assay using the Fast SYBR Green Master Mix (4385612, Thermo Fisher).

### Statistical analysis

Continuous data were expressed in the form of mean ± SD. For 2-group comparisons, the significant difference was determined using Student's *t*-test (unpaired, 2-tailed) and nonparametric Mann-Whitney *U* test for the normally distributed data with equal variance and non-normally distributed data or data with unequal variances respectively. For multi-group comparisons, one-way or two-way analysis of variance (ANOVA) followed by post-hoc Bonferroni's test was used for the normally distributed data with equal variance and Kruskal-Wallis test followed by the post-hoc Bonferroni test for the non-normally distributed data or data with unequal variances. *P* ≤ 0.05 was statistically significant.

## Results

### Diabetic stress leads to increased m^6^A modification level *in vitro* and *in vivo*

**Figure 1:**
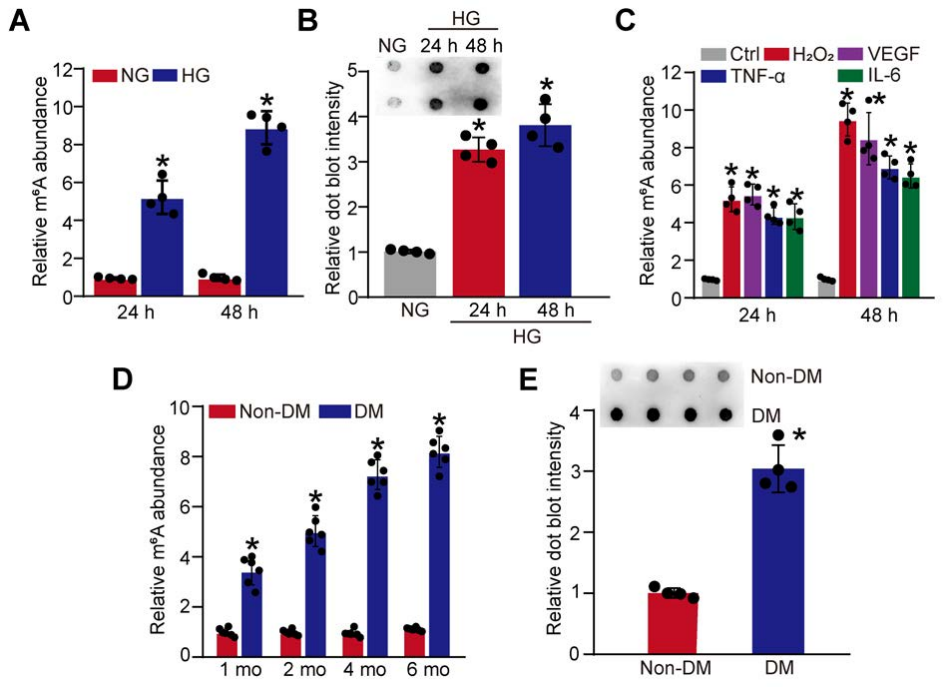
** Diabetic stress leads to increased levels of m^6^A RNA modification *in vitro* and *in vivo.* (A and B)** Pericytes were incubated with 25 mM glucose (High glucose, HG) for 24 h and 48 h. The levels of m^6^A RNAs were detected by the colorimetric quantification (A, n = 4, *P < 0.05 versus NG group, 1-way ANOVA, Bonferroni test) or dot blot assays (B, n = 4, *P < 0.05 versus NG group, 1-way ANOVA, Bonferroni test). **(C)** The levels of m^6^A RNAs were detected by the colorimetric quantification and dot blot assays in pericytes cultured in the medium containing H_2_O_2_ (200 µM), VEGF (10 ng/mL), IL-6 (20 ng/mL), TNF-α (10 ng/mL) or left untreated (Ctrl) for 24 h and 48 h (n = 4, *P < 0.05 versus Ctrl group, 1-way ANOVA, Bonferroni test). **(D and E)** Retinal vessels were isolated from diabetic retinas after 1-month, 2-month, 4-month, and 6-month diabetes induction. The levels of m^6^A RNA modification in retinal vessels were detected by the colorimetric quantification (D, n = 6). After 6-month diabetes induction, dot blot assays were performed to detect the change of m^6^A RNA modification in retinal vessels (E, n = 4). *P < 0.05 versus non-DM group; Mann-Whitney *U* test, Bonferroni test.

Hyperglycemia is a major risk factor of diabetic microvascular complications 20. To determine the relevance of m^6^A RNA modification to retinal pericyte dysfunction under high glucose condition, pericytes were incubated with 25 mM glucose (High glucose, HG) for 24 h and 48 h. Colorimetric quantification and dot blot assays showed that the levels of m^6^A RNA modifications were dramatically increased in pericytes cultured in high glucose medium compared with the control group (Figure 1A-B). Oxidative stress and inflammation are also involved in the process of diabetic microvascular complications 9. The results showed that the levels of m^6^A RNA modifications were also increased in pericytes response to oxidative stress (H_2_O_2_, 200 μM) or inflammatory stimulus, such as VEGF (10 ng/mL), TNF-α (10 ng/mL), and IL-6 (20 ng/mL) (Figure 1C).

We further determined whether diabetic stress altered the levels of m^6^A RNA modification *in vivo*. Retinal vessels were isolated from diabetic mice at 1-month, 2-month, 4-month, and 6-month after the induction of diabetes. Colorimetric quantification and dot blot assay showed that the levels of m^6^A RNA modifications were dramatically increased in diabetic retinal vessels compared with these non-diabetic retinal vessels (Figure 1D-E). Collectively, these results indicate that diabetic stress leads to increased levels of m^6^A RNA modification *in vitro* and *in vivo*.

### Diabetic stress leads to increased METTL3 levels in pericytes and diabetic retinas

**Figure 2:**
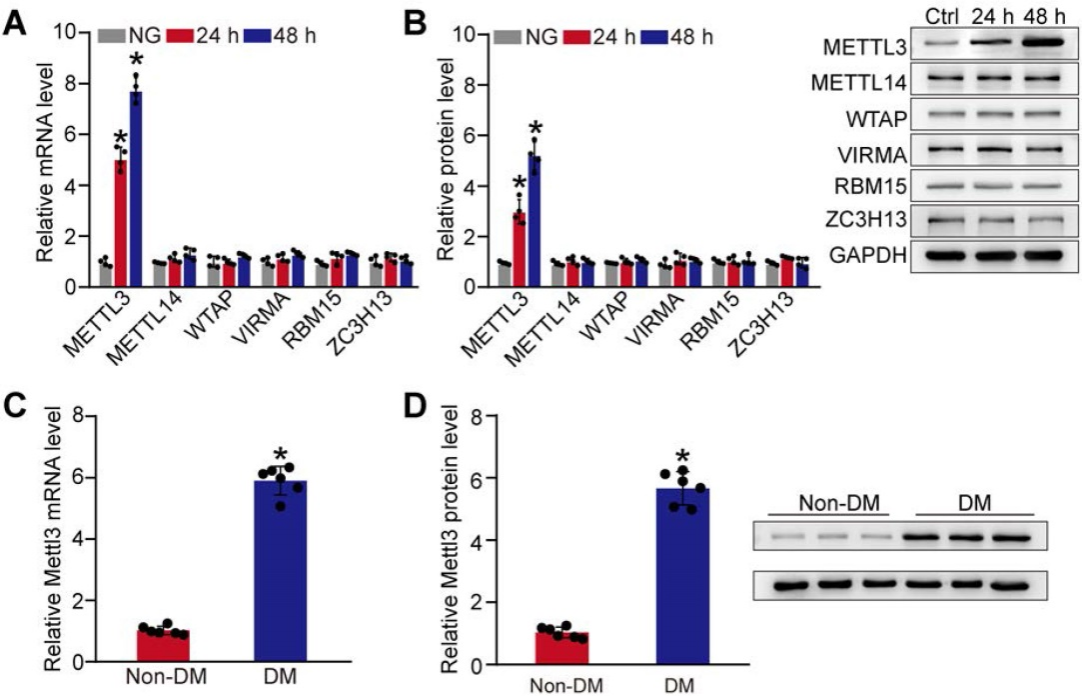
** Diabetic stress induces METTL3 expression in pericytes. (A and B)** Retinal pericytes were incubated with normal culture medium (normal glucose, NG) or 25 mM glucose (high glucose, HG) for 24 h and 48 h. qRT-PCR assays and western blots were conducted to detect the levels of METTL3, METTL14, WTAP, VIRMA, RBM15, and ZC3H13. n = 4; *P < 0.05 versus NG group; 1-way ANOVA, Bonferroni test. **(C and D)** qRT-PCR assays and western blots were conducted to detect the levels of *Mettl3* in the diabetic retinal vessels and the corresponding controls. The representative blots for *Mettl3* were shown. n = 6 retinas per group; *P < 0.05 versus non-DM; Mann-Whitney *U* test, Bonferroni test.

Given that m^6^A RNA modification is mediated by m^6^A methyltransferases, such as METTL3, METTL14, WTAP, VIRMA, RBM15, and ZC3H13 21, we then detected the expression pattern of these regulators at the mRNA and protein level in pericytes. Compared with the control group, qRT-PCRs and western blots revealed that the levels of METTL3 were significantly increased in pericytes response to high glucose stress both at mRNA and protein levels. By contrast, the levels of METTL14, WTAP, VIRMA, RBM15, or ZC3H13 were not altered response to high glucose stress (Figure 2A-B). We also compared the expression pattern of *Mettl3* between diabetic retinas and normal retinas. Compared to normal retinas, diabetes led to increased levels of *Mettl3* in the retinas both at mRNA and protein levels (Figure 2C-D). Collectively, these results suggest that METTL3 is involved in the regulation of mRNA m^6^A modification under diabetic stress.

### METTL3 regulates retinal pericyte dysfunction *in vitro*

**Figure 3:**
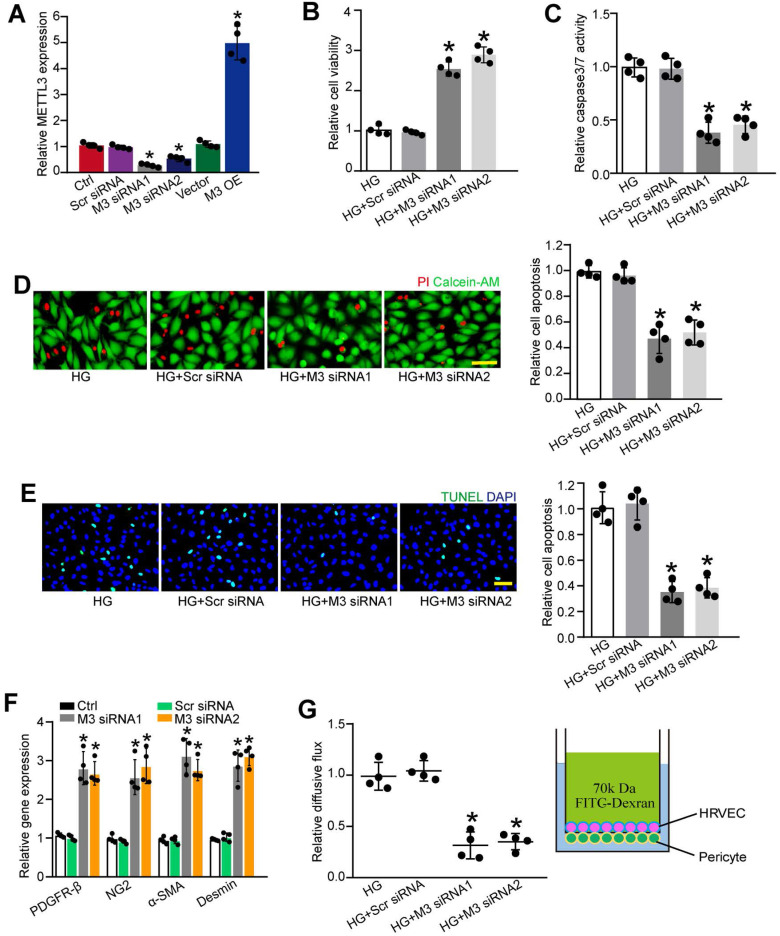
** METTL3 silencing suppresses pericyte dysfunction *in vitro.* (A)** Retinal pericytes were transfected with scrambled (Scr) siRNA, METTL3 siRNA1 (M3 siRNA1), METTL3 siRNA2 (M3 siRNA2), pcDNA 3.1 vector (Vector), pcDNA 3.1-METTL3 (M3 OE), or left untreated (Ctrl) for 48 h. qRT-PCR assays were conducted to detect the levels of METTL3 (A, n = 4, *P < 0.05 versus Ctrl group; Student's *t*-test).** (B-D)** Pericytes were transfected with Scr siRNA, M3 siRNA1, M3 siRNA2, or left untreated, and then exposed to 25 mM glucose for 48 h. MTT assays were conducted to detect cell viability (**B,** n = 4, *P < 0.05 versus HG group;1-way ANOVA, Bonferroni test). Apoptotic cells were determined by caspase 3/7 activity (**C,** n=4, *P < 0.05 versus HG group; 1-way ANOVA, Bonferroni test), Calcein-AM/PI staining (**D,** n = 4, Scale bar: 20 µm, *P < 0.05 versus HG group; 1-way ANOVA, Bonferroni test), and TUNEL staining (**E,** n = 4, Scale bar: 20 µm, *P < 0.05 versus HG group; 1-way ANOVA, Bonferroni test). The expression levels of pericyte markers, including PDGFR-β, NG2, α-SMA, and Desmin were determined by qRT-PCRs in the pericytes after transfection of Scr siRNA, M3 siRNA1, M3 siRNA2, or left untreated (Ctrl) for 48 h (**F,** n = 4, *P < 0.05 versus Ctrl group;1-way ANOVA, Bonferroni test). The barrier function was analyzed by the measurement of 70-kDa FITC-Dextran leakage. The treated pericytes were co-cultured with endothelial cells in the insert chambers and relative diffusive flux change was used to show the barrier permeability (**G,** n = 4, *P < 0.05 versus HG group; 1-way ANOVA, Bonferroni test).

To determine the role of METTL3 in pericytes upon high glucose stress, we silenced METTL3 expression in pericytes using METTL3 small interfering RNAs (siRNAs) or up-regulated the level of METTL3 by the gain-of-function of METTL3. qRT-PCR assays showed that the transfection of METTL3 siRNA led to decreased levels of METTL3. By contrast, METTL3 overexpression led to increased levels of METTL3 (Figure 3A). Moreover, the transfection of METTL3 siRNA significantly reduced m^6^A levels, but the gain-of-function of METTL3 increased m^6^A levels both under normal condition and high glucose condition (Figure S1), indicating that METTL3 regulates m^6^A RNA methylation in pericytes.

We then determined that the role of METTL3 in cell viability and apoptosis under diabetic condition. Pericytes were exposed to high glucose to mimic diabetic condition *in vitro*. MTT (3-[4, 5-Dimethythiazol-2-yl]-2, 5-diphenyl tetrazolium bromide) assays showed that compared with HG group, METTL3 silencing increased the viability of pericytes under high glucose stress (Figure 3B). Caspase 3/7 activity assays, Calcein-AM/propidium iodide (PI) staining, and TUNEL staining showed that the transfection of METTL3 siRNA could reduce high glucose stress-induced pericyte apoptosis as shown by decreased caspase 3/7 activity (Figure 3C), decreased number of PI-positive cells (Figure 3D), and decreased number of TUNEL-positive cells (Figure 3E). Normal pericyte function is important for the maturation of retinal vessels and the stability of blood-retina barrier 14. METTL3 silencing by METTL3 siRNA1 or siRNA2 led to increased expression of pericyte markers, including platelet-derived growth factor receptor (PDGFR)-β, α-SMA, Desmin, and NG2 (Figure 3F). In the co-culture model of pericytes and HRVECs, METTL3 silencing in pericytes significantly reduced the permeability of macromolecules compared with HG group (Figure 3G).

To further explore the role of m^6^A RNA modification in retinal pericytes, we determined the effects of the gain-of-function of METTL3 in pericytes. METTL3 overexpression aggravated high glucose-induced pericyte dysfunctoin as shown by decreased viability, increased permeability of macromolecules, and increased PI-positive cells (Figure S2). Collectively, these results indicate that METTL3 regulates pericyte dysfunction *in vitro*.

### Pericyte-specific deletion of *Mettl3* alleviates pericyte dysfunction and retinal vascular complication *in vivo*

**Figure 4:**
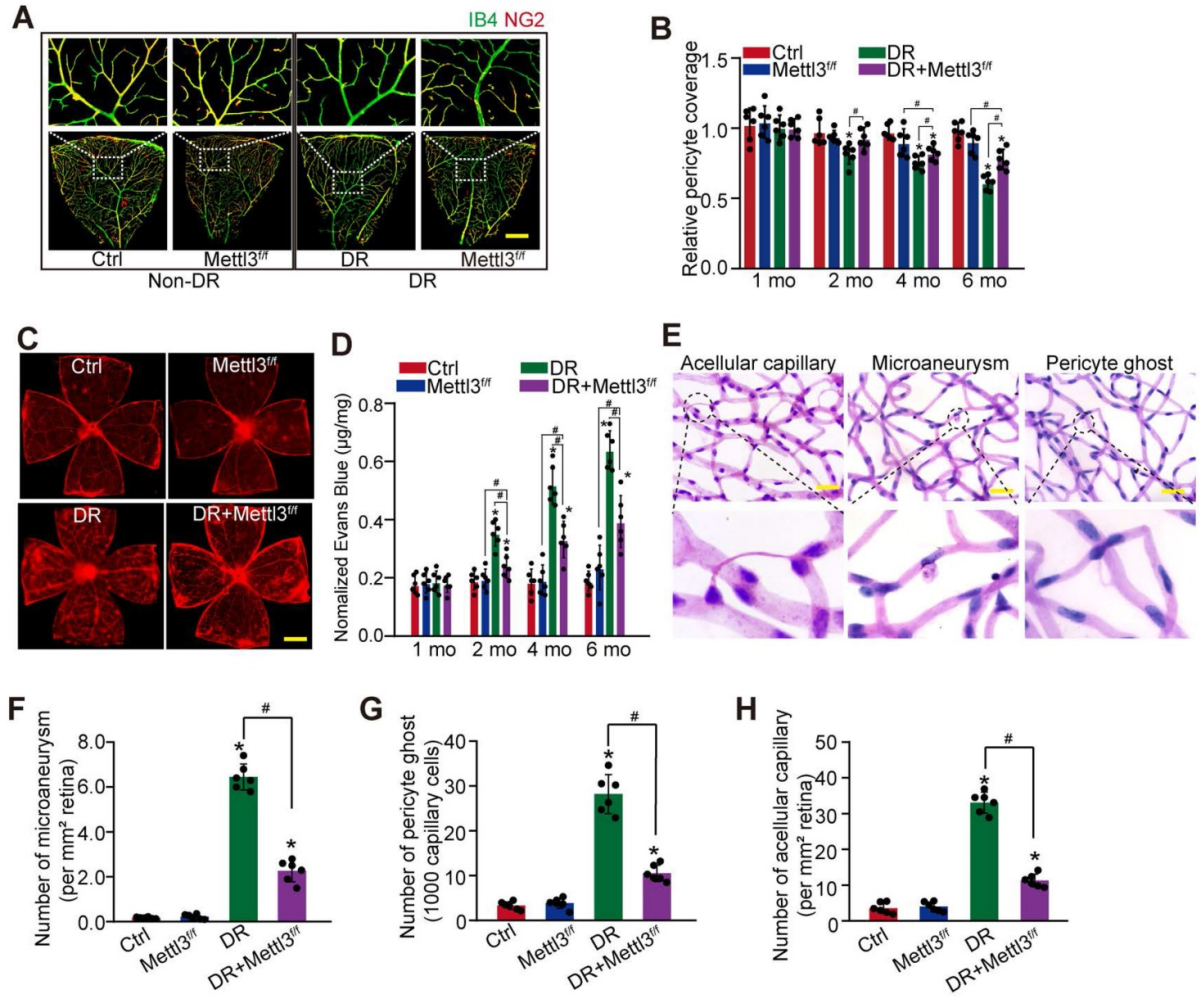
** Pericyte-specific deletion of *Mettl3* alleviates pericyte dysfunction and retinal vascular complication *in vivo.* (A and B)** *Mettl3^f/f^; Pdgfrβ-Cre* (Mettl3^f/f^) mice or *Mettl3^+/+^; Pdgfrβ-Cre* (*Mettl3*^+/+^) mice (Ctrl group) were intraperitoneally injected with STZ for diabetes induction. The flat-mounted retinas were stained by IB4 (in green, endothelial cells) and NG2 (in red, pericytes) to detect pericyte coverage on retinal vessels (n = 6 retinas per group, Scale bar: 100 µm). The multiple overlapping images of flat-mounted retinas were captured by a ×10 lens and the individual images were arrayed to obtain the composite images of a leaf of retina vessels. The representative composite images after 6 months of treatment and statistical result was shown. **(C and D)** Evans blue assay was conducted to detect retinal vascular leakage. Evans blue dye was injected and circulated for 2 h. After fixation, the whole flat-mounted retinas were tiled and observed under a fluorescence microscope at a × 4 lens to take tile-scanning images. The representative composite images of flat-mounted retinas and the quantification results were shown. The red fluorescent is Evans blue signaling (n = 6 retinas per group, Scale bar: 500 µm). **(E-H)** Retinal trypsin digestion was conducted to detect the number of microaneurysms (F, n = 6 retinas per group, per mm^2^ retina), pericyte ghosts (G, n = 6 retinas per group, per 1000 capillary cells), and acellular capillaries (F, n = 6 retinas per group, per mm^2^ retina). The representative images of acellular capillary, pericyte ghost, and microaneurysm were shown (E, Scale bar: 100 µm). *P < 0.05 versus Ctrl group;^ #^*P* < 0.05 between the marked groups; Kruskal-Wallis test, Bonferroni test.

To reveal the role of *Mettl3* in pericytes *in vivo*, pericyte-specific deletion of *Mettl3* mice (*Mettl3*^f/f^; *Pdgfrβ*-Cre mice) were generated using the Cre/LoxP system. The deletion efficiency was determined by western blots. Compared with Ctrl group, the levels of* Mettl3* in the primary pericytes isolated from *Mettl3*^f/f^; *Pdgfrβ*-Cre mice (*Mettl3*^f/f^ group) were significantly reduced (Figure S3). IB4 and NG2 immunofluorescence staining showed pericyte-specific deletion of *Mettl3* significantly decreased diabetes-induced pericyte dysfunction in *Mettl3*^f/f^; *Pdgfrβ*-Cre mice compared with the control group (Figure 4A-B). Pericyte dropout from retinal capillaries can induce retinal vascular leakage. Evans blue assay showed that pericyte-specific deletion of *Mettl3* reduced diabetes-induced retinal vascular leakage (Figure 4C-D). Pericyte dropout can evoke retinal microaneurysms and acellular capillary formation 9. Trypsin digest and periodic acid Schiff (PAS) staining revealed that pericyte-specific deletion of *Mettl3* significantly decreased the number of microaneurysms, acellular capillaries, and pericyte ghosts in diabetic retinas (Figure 4E-H). These results suggest that pericyte-specific deletion of *Mettl3* can protect against diabetes-induced retinal vascular dysfunction *in vivo*.

### Identification of the potential targets of METTL3 in pericytes

**Figure 5:**
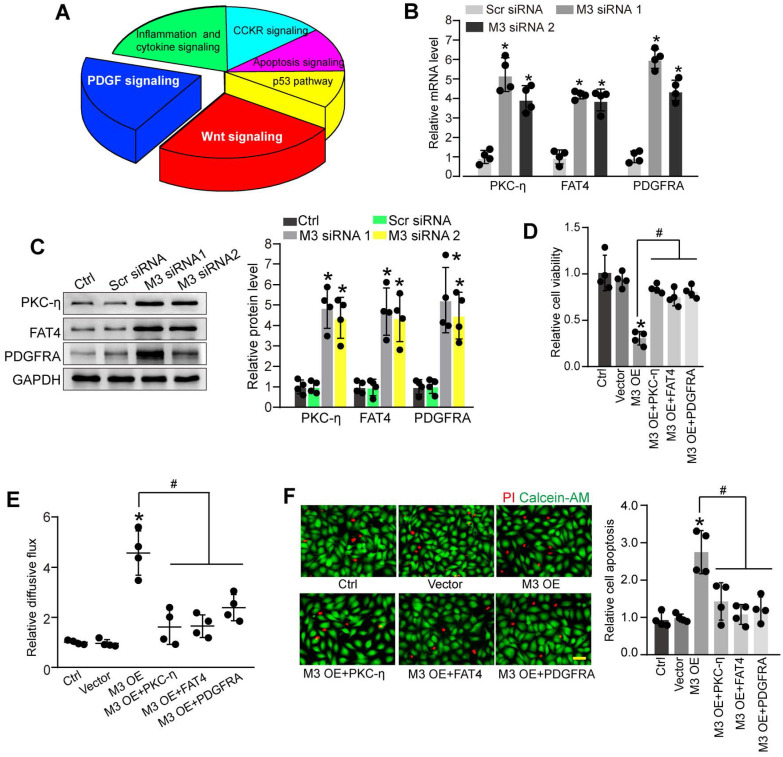
** Identification of the potential targets of METTL3 in pericytes. (A)** KEGG pathway analysis was conducted to predict the potential signaling pathways regulated by METTL3 in pericytes. **(B and C)** Pericytes were transfected with scrambled siRNA (Scr siRNA), METTL3 siRNA1 (M3 siRNA1), METTL3 siRNA2 (M3 siRNA2), or left untreated (Ctrl) for 48 h. qRT-PCRs were conducted to detect the expression of PKC-η, FAT4, and PDGFRA in pericytes (**B,** n = 4, *P < 0.05 versus Ctrl group). Western blots were conducted to detect the levels of PKC-η, FAT4, and PDGFRA in pericytes. Representative blots and statistical results were shown (**C,** n = 4, *P < 0.05 versus Ctrl group). **(D-F)** Retinal pericytes were transfected with pcDNA 3.1 vector (Vector), pcDNA 3.1-METTL3 (M3 OE) without or with PKC-η, FAT4, or PDGFRA overexpression, or left untreated (Ctrl). These cells were exposed to 25 mM glucose for 48 h. MTT assays were conducted to detect cell viability (**D,** n = 4). The endothelial permeability was measured by FITC-Dextran transwell assay (**E,** n = 4). Apoptotic cells were determined by Calcein-AM/PI staining. Representative PI staining images at 48 h and statistical result were shown (**F,** n = 4, Scale bar: 20 µm). *P < 0.05 versus Ctrl group;^ #^*P* < 0.05 versus M3 OE group; 1-way ANOVA, Bonferroni test.

To further explore the molecular mechanism of METTL3-mediated pericyte dysfunction, the pericytes were transfected with METTL3 siRNA for 48 h to decrease METTL3 levels. We compared the whole transcriptional profiling between normal pericytes and METTL3 silencing pericytes by performing RNA sequencing (RNA-Seq). The results showed that 872 differentially expressed genes were identified, including 790 up-regulated genes and 82 down-regulated genes (Table S1). GO analysis demonstrated that the significant enriched GO term in cellular component was protein-containing complex. The enriched GO term in biologic process was cellular process. The most enriched GO term in molecular function was catalytic activity (Figure S4). KEGG pathway analysis revealed these differentially expressed genes were significantly enriched in Wnt signaling, PDGF signaling, inflammation and cytokine signaling, CCKR signaling, Apoptosis signaling, and p53 signaling. Notably, Wnt signaling and PDGF signaling were the ranked as the Top 2 enriched signaling pathways (Figure 5A).

PKC-η, FAT4, and PDGFRA are important members involved in Wnt signaling and PDGF signaling pathway 22, 23, which have been identified as the differentially expressed genes between normal pericytes and METTL3 silencing pericytes in RNA-Seq assays. qRT-PCR assays and western blots showed that METTL3 silencing led to increased expression levels of PKC-η, FAT4, and PDGFRA in pericytes (Figure 5B-C). By contrast, METTL3 overexpression reduced PKC-η, FAT4, and PDGFRA expression both at mRNA and protein levels (Figure S5A-B). In particular, the m^6^A abundance of PKC-η, FAT4, and PDGFRA was markedly decreased upon METTL3 silencing as shown by gene specific m^6^A-qPCRs (Figure S5C), suggesting PKC-η, FAT4, and PDGFRA are the potential targets of METTL3.

We further investigated whether PKC-η, FAT4, or PDGFRA overexpression could rescue the effects of METTL3 overexpression on pericyte function *in vitro*. PKC-η, FAT4, or PDGFRA overexpression could partially alleviate METTL3 overexpression-induced viability reduction of pericytes (Figure 5D). Co-culture assays showed that METTL3 overexpression in pericytes led to increased permeability of macromolecules compared with the control group. By contrast, PKC-η, FAT4, or PDGFRA overexpression could partially reduce the permeability of macromolecules (Figure 5E). Under high glucose condition, the overexpression of PKC-η, FAT4, or PDGFRA could alleviate METTL3 overexpression-induced apoptosis of pericytes (Figure 5F).

### METTL3-PKC-η/FAT4/PDGFRA axis regulates pericyte dysfunction and retinal vascular complication *in vivo*

**Figure 6:**
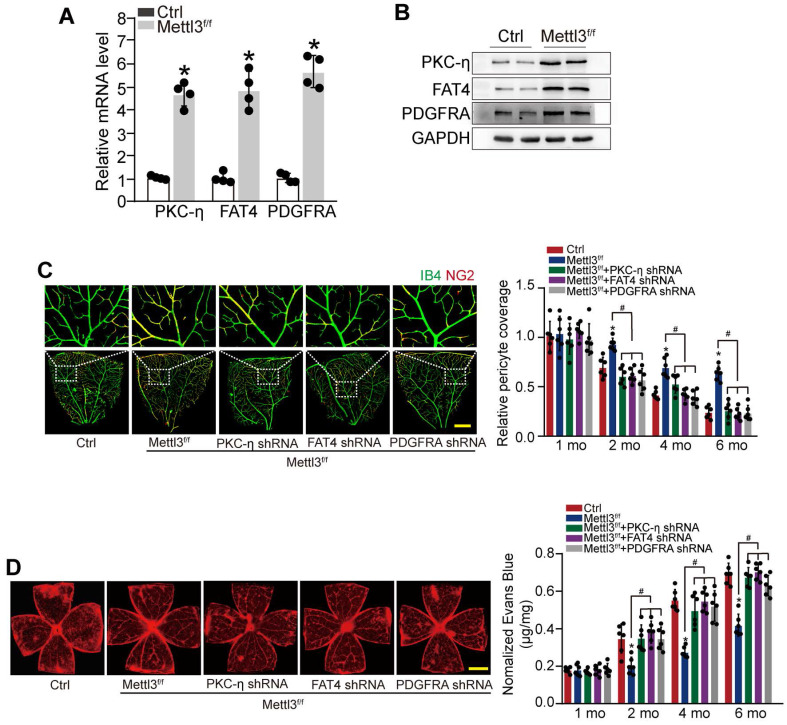
** METTL3-PKC-η/FAT4/PDGFRA axis regulates pericyte dysfunction and retinal vascular complication *in vivo.* (A and B)** qRT-PCRs (A, n = 4 retinas per group) and western blots (B, n = 4 retinas per group) were conducted to detect the expression of PKC-η, FAT4, and PDGFRA in pericyte-specific* Mettl3* deletion mice (*Mettl*3^f/f^; *Pdgfrβ*-Cre mice) (Mettl3^f/f^) and *Mettl*3^+/+^ mice. The representative dots were shown. **(C and D)** Diabetic mice (Ctrl) or diabetic pericyte-specific *Mettl3* deletion mice received an intravitreous injection of PKC-η shRNA, FAT4 shRNA or PDGFRA shRNA, or were left untreated. Pericyte coverage was quantified by staining the whole-mount retinas with IB4 and NG2 after 1 month, 2 months, 4 months, and 6 months of treatment (**C,** n = 6 retinas per group, Scale bar: 100 µm). Evans blue assay was conducted to detect retinal vascular leakage. Evans blue dye was injected and circulated for 2 h. After the fixation, the whole flat-mounted retinas were tiled and observed under a fluorescence microscope at a × 4 lens to take tile-scanning images. The representative composite images of flat-mounted retinas and the quantification results were shown. The red fluorescent is Evans blue signaling (n = 6 retinas per group, Scale bar: 500 µm). *P < 0.05 versus Ctrl group, #*P* < 0.05 among the marked groups. Kruskal-Wallis test, Bonferroni test.

To reveal the role of METTL3-PKC-η/FAT4/PDGFRA axis in pericyte dysfunction *in vivo*, pericyte-specific* Mettl3* deletion mice (*Mettl*3^f/f^; *Pdgfrβ*-Cre mice) was generated using the Cre/LoxP system. qRT-PCRs and western blots showed that pericyte-specific* Mettl3* deletion led to increased expression of PKC-η, FAT4, and PDGFRA (Figure 6A-B). IB4 and NG2 immunofluorescence staining showed that pericyte-specific deletion of *Mettl3* led to increased pericyte coverage of retinal capillaries and reduced retinal vascular leakage under diabetic stress. However, silencing of PKC-η, FAT4, or PDGFRA could partially reduce the protective effects of *Mettl3* deletion on retinal vascular complication as shown by reduced pericyte coverage and increased vascular leakage under diabetic stress (Figure 6C-D).

### METTL3 regulates the stability of PKC-η, FAT4, and PDGFRA mRNA in YTHDF2-dependent manner

**Figure 7:**
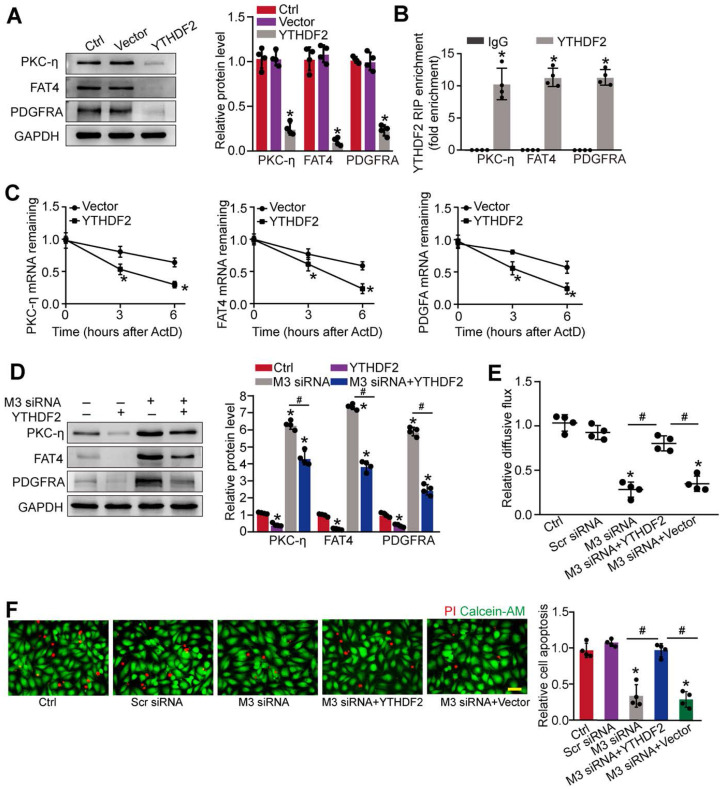
** METTL3 regulates the stability of PKC-η, FAT4, and PDGFRA mRNA in YTHDF2-dependent manner. (A)** Pericytes were transfected with pcDNA3.1-YTHDF2 (YTHDF2), pcDNA3.1 vector (Vector), or left untreated (Ctrl) for 48 h. Western blots were conducted to detect the expression of PKC-η, FAT4, or PDGFRA. Representative blots and quantification result was shown (n = 4, *P < 0.05 versus Ctrl group, 1-way ANOVA, Bonferroni test). **(B)** RNA pull-down assay was conducted to confirm the association between YTHDF2 and PKC-η, FAT4, or PDGFRA. The levels of PKC-η, FAT4, and PDGFRA were detected by qRT-PCR assays (n = 4, *P < 0.05 versus IgG group, Student *t* test). **(C)** qRT-PCR assays were conducted to detect the levels of PKC-η, FAT4, and PDGFRA mRNA in pericytes overexpressing pcDNA3.1-YTHDF2 (YTHDF2) or pcDNA3.1 vector (Vector) at indicated time after the treatment of transcription inhibitor, actinomycin D (*P < 0.05 versus Vector group, n = 4, 1-way ANOVA, Bonferroni test). **(D)** The pericytes were treated as shown. Western blots were conducted to detect the expression of PKC-η, FAT4, and PDGFRA. Representative immunoblots and quantification results were shown (n = 4, *P < 0.05 versus Ctrl group, *#P* < 0.05 M3 siRNA group+YTHDF2 versus M3 siRNA group, 1-way ANOVA, Bonferroni test). **(E and F)** Pericytes were transfected with Scr siRNA, M3 siRNA, M3 siRNA+YTHDF2, vector, or left untreated (Ctrl), and then exposed to 25 mM glucose for 48 h. FITC-Dextran assay was conducted to evaluate the barrier function (E, n = 4, 1-way ANOVA, Bonferroni test). Apoptotic cells were determined by PI staining (F, n = 4, Scale bar: 20 µm). *P < 0.05 versus Ctrl group;^ #^*P* < 0.05 between the marked groups; 1-way ANOVA, Bonferroni test.

We next investigated the underlying mechanism of PKC-η, FAT4, and PDGFRA expression repression by m^6^A modification. Previous studies have shown that the function of m^6^A modification in mRNA metabolism depends on the reader proteins, including YT521-B homology (YTH) domain family (YTHDF1-3, YTHDC1-2), and IGF2 mRNA binding protein family 24. To determine which reader protein is responsible for m^6^A modification in pericytes, YTHDF1-3, YTHDC1-3, or IGF2 was overexpressed in pericytes. Up-regulation of YTHDF2, but not other readers led to decreased expression of PKC-η, FAT4, and PDGFRA in pericytes (Figure 7A and Figure S6A-F). By contrast, silencing of YTHDF2 led to increased protein levels of PKC-η, FAT4, and PDGFRA (Figure S6G). As expected, RIP-qPCR analysis revealed that the levels of PKC-η, FAT4, and PDGFRA were enriched in the immunoprecipitates pulled down by YTHDF2 but not IgG (Figure 7B). To determine whether YTHDF2 affected PKC-η, FAT4, and PDGFRA expression through regulating mRNA decay, mRNA levels were detected after the treatment of transcription inhibitor, actinomycin D. The half-life of PKC-η, FAT4, or PDGFRA mRNA was significantly reduced in the pericytes overexpressing YTHDF2 compared to control group (Figure 7C). In pericytes, METTL3 silencing led to increased expression of PKC-η, FAT4, and PDGFRA, which could be partially repressed by YTHDF2 overexpression (Figure 7D). In pericyte-endothelial cell co-culture experiment, METTL3 silencing in pericytes decreased the permeability of macromolecules compared with the control group. By contrast, YTHDF2 overexpression led to increased permeability of macromolecules (Figure 7E). Under high glucose condition, YTHDF2 overexpression could abolish the protective effects of METTL3 silencing on pericyte survival (Figure 7F).

## Discussion

*N*^6^-methyladenosine (m^6^A) has shown as the critical regulator of the modified mRNAs 25. Herein, we show that the total levels of m^6^A RNAs are markedly increased in retinal pericytes upon diabetic stress. Decreased m^6^A methylation suppresses diabetes-induced retinal pericyte dysfunction and alleviates retinal vascular complication. By contrast, increased m^6^A methylation aggravates pericyte dysfunction and accelerates retinal vascular complication, suggesting that m^6^A methylation acts as an intrinsic regulator of pericyte biology.

m^6^A RNA methylation is the most prevalent internal RNA modification. Its homeostasis is maintained through the coordinated regulation of m^6^A methylases and demethylases 21, 26. We show that diabetic stress leads to increased METTL3 level, but has no effect on the expression of other m^6^A methylases and demethylases in pericytes. Increased METTL3 accelerates pericyte apoptosis and decreases pericyte viability. Under diabetic stress, METTL3 silencing can protect pericytes against diabetes-induced pericyte dysfunction and alleviate BRB breakdown *in vitro*. Moreover, conditional pericyte knockout of *Mettl3* can reduce pericyte loss and diabetes-induced retinal vascular complications. Thus, silencing of METTL3 can become a promising strategy for the treatment of diabetes-induced retinal vascular complications.

Retinal vessels are mainly composed of pericytes and endothelial cells (ECs). Pericytes provide vascular stability and control endothelial proliferation. Pericyte loss, microaneurysms, and acellular capillaries are the important pathological features of diabetic retinal vessels 9, 27. At the early stage of DR, pericyte dysfunction is shown as the earliest histopathological hallmark 28. METTL3 silencing *in vitro* or conditional knockout of METTL3 *in vivo* protects pericytes against diabetes-induced injury. At the proliferative stage of DR, retinal neovascularization is caused by abnormal activation of ECs 29. However, the asynchronized or insufficient proliferation and recruitment of pericytes affects the maturation of new blood vessels. In our previous studies, we have revealed that conditional knockout of METTL3 in ECs inhibits abnormal activation of ECs and pathological angiogenesis 30. Herein, we show that specific knockout of METTL3 in pericytes can reduce pericyte dysfunction via affecting their proliferation, viability, and differentiation, which might contribute to the maturation of new blood vessels. Due to the critical role of METTL3 in pericytes and ECs, it is not surprising that METTL3 is involved in diabetes-induced retinal vascular complications.

RNA-Seq analysis has revealed that 872 genes are dysregulated in METTL3 silencing pericytes. KEGG pathway analysis reveals that these dysregulated genes are enriched in Wnt signaling and PDGFR signaling. Moreover, the members of Wnt signaling and PDGFR signaling including PKC-η, FAT4, and PDGFRA are significantly up-regulated upon METTL3 silencing. FAT4 can encode cadherin protein. Ablation of cadherin impairs pericyte recruitment and affects pericytic-endothelial interaction 31, 32. PDGFRA encodes a cell surface tyrosine kinase receptor for members of PDGF family. These growth factors are mitogens for cells, which plays a role in organ development, wound healing, and tumor progression 33. PKCη is a member of the novel PKC isozymes that regulates cell proliferation, differentiation, secretion and apoptosis 34, 35. It can phosphorylate several protein targets and is involved in many signaling pathways, such as Wnt signaling and PDGF signaling. During the progression of DR, high glucose leads to increased levels of METTL3. Increased METTL3 level reduces the expression of PKC-η, FAT4, and PDGFRA. Due to the critical role of PKC-η, FAT4, and PDGFRA in pericyte functions, it is not surprising that METTL3-mediated signaling is involved in the regulation of pericyte dysfunction.

m^6^A modification plays important roles in several aspects of gene expression 15. They can also influence the fate of mRNAs via m^6^A reader proteins. Several YTH domain-containing proteins have been identified as m^6^A readers, such as YTHDF2, YTHDF1, YTHDF3, and YTHDC1 18. Increased METTL3 leads to a marked reduction of PKC-η, FAT4, and PDGFRA level, which mainly derives from the involvement of m^6^A reader protein, YTHDF2. YTHDF2 binds to the m^6^A-modified mRNAs of PKC-η, FAT4, and PDGFRA and induces their degradation. Reduced expression of PKC-η, FAT4, and PDGFRA would affect the proliferation, differentiation, and differentiation of pericytes. By contrast, METTL3 silencing decreases the decayed amount of PKC-η, FAT4, and PDGFRA, contributing to improving pericyte dysfunction. Thus, METTL3 triggers RNA modification on its downstream mRNA transcripts and orchestrates YTHDF2 to determine the fates of PKC-η, FAT4, and PDGFRA.

## Conclusions

This study uncovers a critical role of METTL3-dependent m^6^A methylation in directing pericyte dysfunction. METTL3 overexpression impairs pericyte differentiation, proliferation, and differentiation *in vitro*. Conditional ablation of METTL3 in pericytes suppresses diabetes-induced retinal pericyte loss, vascular leakage, and vascular lesions *in vivo*. METTL3 directs m^6^A modification of PKC-η, FAT4, and PDGFRA mRNA to induce mRNA decay via YTHDF2-dependent pathway. Together, this study unveils the functional importance of m^6^A methylation machinery in pericyte biology, which expands the understanding of such interplay that is essential for development of therapeutic strategies for the prevention and treatment of diabetic microvascular complications.

## Supplementary Material

Supplementary methods and figures.

Supplementary table.
