# Supplementary material for: METTL3-mediated N6-methyladenosine modification governs pericyte dysfunction during diabetes-induced retinal vascular complication
Source: Theranostics. 2022 Jan 1;12(1):277–89. doi: 10.7150/thno.63441 (PMC8690932; doi:10.7150/thno.63441)
Supplement: Supplementary file 1 — Supplementary methods and figures. [file thnov12p0277s1.pdf]

Supplementary Information for

# METTL3-Mediated $N^6$-Methyladenosine Modification Governs Pericyte Dysfunction during Diabetes-Induced Retinal Vascular Complication

**This PDF file includes:**

Supplementary text

Figs. S1 to S6

# Supplemental information text

## Evans blue assay

Evans blue (EB) dye was used to detect retinal vascular permeability. Briefly, the mice were anesthetized by intraperitoneal injections of ketamine (80 mg/kg) and xylazine (4 mg/kg). EB dye (100 mg/ml, E2129, Sigma, USA) was dissolved in normal saline, sonicated for 5 min in an ultrasonic cleaner (G1125P1T, Laboratory Supplies, USA), and filtered by a 0.22-μm filter (Millipore, USA). EB was injected through the femoral vein over 10 sec at a dosage of 45 mg/kg and the mice were kept on a warm pad for 2 h. The retina was isolated after the perfusion with normal saline and fixed in 4% PFA for 30 min. Then, the retina was flat-mounted and photograph under a fluorescence microscope (IX73P1F, Olympus, Japan) to detect EB extravasation from retinal vessels. For quantitative analysis of vascular permeability, the blood samples were obtained from the iliac artery to determine the concentration of EB dye in the plasma. After the measurement of retinal wet weight, the retina was thoroughly dried in a Speed-Vac. EB dye was extracted by incubating each retina in 120 μL formamide for 18 h at 70°C. The extract was ultra-centrifuged at a speed of 70,000 rpm for 45 min at 4°C. Spectrophotometric method was used to detect the supernatant absorbance at 620 nm (blue signal) and 740 nm (background subtracted). The concentration of dye in the extracts was calculated from the standard curve of EB in formamide. After the centrifugation at 3,600 g for 15 min at 25°C, the concentration of EB dye in blood sample was measured in the same way. Vascular permeability was calculated using the equation listed below:

$$\frac{\text{Retinal Evans blue } (\mu g) / \text{ Retinal dry weight } (mg)}{\text{Time averaged Evans blue concentration } (\mu g)/\text{Plasma } (\mu l) \times \text{Circulation time } (h)}$$

**RNA isolation and quantitative reverse transcription PCR (qRT-PCR)**

TRIzol (15596-026, Invitrogen, USA) was used to extract the total RNAs according to the manufacturer's instruction. The concentration of each sample was examined by a spectrophotometer at 260 nm. cDNAs were synthesized using 1 μg of total RNAs by the PrimeScript RT Master Mix (RR036A, Takara, Japan). The gene expression level was detected by qRT-PCRs using the SYBR Premix Ex Taq II (RR820A, Takara, Japan). The cycling conditions were conducted as follows: initial denaturation at 95°C for 3 min, followed by 40 cycles of 1 min denaturation at 95°C, 30 sec primer annealing at 55°C, and 45 sec extension at 72°C followed by a final extension at 72°C for 5 min. Relative gene expression was normalized to GAPDH mRNA expression using the $2^{-\Delta\Delta Ct}$ method. All used primers were listed as below:

| | | |
|---|---|---|
| GAPDH | Forward<br>Reverse | 5′-AAGACGGGCGGAGAGAAACC-3′<br>5′-CGTTGACTCCGACCTTCACC-3′ |
| *Gapdh* | Forward<br>Reverse | 5′-AGGTCGGTGTGAACGGATTTG-3′<br>5′-TGTAGACCATGTAGTTGAGGTCA-3′ |
| METTL3 | Forward<br>Reverse | 5′-TGATTGAGGTAAAGCGAGGTC-3′<br>5′-TCCTGACTGACCTTCTTGCTC-3′ |
| *Mettl3* | Forward<br>Reverse | 5′-GGGCACTTGGATTTAAGGAACC-3′<br>5′-CTTAGGGCCGCTAGAGGTAGG-3′ |
| METTL14 | Forward<br>Reverse | 5′-AGAAACTTGCAGGGCTTCCT-3′<br>5′-TCTTCTTCATATGGCAAATTTTCTT-3′ |
| WTAP | Forward<br>Reverse | 5′-TTCCCAAGAAGGTTCGATTG-3′<br>5′-TGCAGACTCCTGCTGTTGTT-3′ |
| RBM15 | Forward<br>Reverse | 5′-ACCGCAGTCCAGAATTGAGC-3′<br>5′-ACTTCAGCTTGGAGGAAGCAG-3′ |
| ZC3H13 | Forward<br>Reverse | 5’-GTGCCGTAACTGGCTGAAGA-3’<br>5’-CCTTTACCACGAGGTGAAGGG-3’ |
| VIRAM | Forward<br>Reverse | 5'-CTTGGCAAGTGGCTTGAACC-3'<br>5'-ACGTAAGGCAGTGGTAAGGC-3' |

| | | |
|---|---|---|
| PDGFR-β | Forward<br>Reverse | 5'-TGGTGCTCACCATCATCTCC-3'<br>5'-CACCTTCCATCGGATCTCGTAA-3' |
| NG2 | Forward<br>Reverse | 5'-GCTTTGACCCTGACTATGTTGGC-3'<br>5'-TCCAGAGTAGAGCTGCAGCA-3' |
| Desmin | Forward<br>Reverse | 5'-TGAAGGGCACTAACGATTCC-3'<br>5'-CTCAGAACCCCTTTGCTCAG-3' |
| α-SMA | Forward<br>Reverse | 5'-ACTGAGCGTGGCTATTCCTCCGTT-3'<br>5'-GCAGTGGCCATCTCATTTTCA-3' |

**TUNEL staining**

The apoptosis of pericytes was determined by the terminal dUTP transferase nick end labeling (TUNEL) staining (C1088, Beyotime). Briefly, after the required treatment, the pericytes were fixed with 4% paraformaldehyde for 30 min at room temperature, washed thrice with PBS, and permeabilized with 0.1% Triton X-100 for 5 min at room temperature. Then, the pericytes were stained with 50 μl TUNEL reaction mixture at 37°C for 60 min and washed with PBS. Finally, the nuclei were stained with DAPI (1:1500, C1002, Beyotime). Images were acquired under using a fluorescence microscope (IX73P1F, Olympus, Tokyo, Japan).

**Isolation of retinal vessels**

The whole eyes from C57BL/6J mice were resected and then the retina was gently separated and cut from the anterior portion of the eyeball. The retinas were firstly rinsed three times in ice-cold MEM D-Valine with HEPES buffer and pooled in the same solution. To obtain the retinal microvessels, the retinas were enzymatically digested in collagenase/dispase (1 mg/mL; Roche, UK) and DNase (20 U/mL, Ambion, USA) at 37°C for 45 min. The microvessels were extracted using anti-mouse PECAM-1 antibodies, followed by sheep anti-mouse IgG-conjugated magnetic beads (Dynabead; Invitrogen, USA) and magnetic separation. The bead-bound vessels

(vessel-enriched) and vessel-depleted fractions were separately lysed for subsequent analysis. Ten retinas were pooled together as a group.

**FITC-Dextran transwell assay**

After the required treatment, pericytes were planted onto the abluminal side of the transwell filter (0.4 μm pore size, polycarbonate membrane, Corning, USA) pre-coated with fibronectin (30 μg/mL) and cultured to form a confluent monolayer. Then, HRVECs were seeded onto the luminal side and co-cultured with pericytes. Culture medium was changed every other day. FITC-conjugated dextran (70kDa, 0.25 mg/mL, #FD70S-100MG, Sigma-Aldrich; USA) was added to the upper compartment and the aliquots were collected at 1, 2, 3 and 4 h after the addition. A sample was taken from the lower compartment and the fluorescent signal was detected using a fluorescent plate reader. The concentration of FITC-dextran was calculated using an experimentally derived standard curve and the rate of diffusive flux (*Po*) was calculated using the following formula:

$$Po = [(F_A/\Delta t)\ V_A] / (F_L\, A)$$

where, $Po$ is diffusive flux; $F_A$ is basolateral fluorescence; $F_L$ is apical fluorescence; $\Delta t$ is change in time; $A$ is the surface area of the filter (in square centimeters); and $V_A$ is the volume of the basolateral chamber (in cubic centimeters).

**Calcein-AM/propidium iodide (PI) double staining**

After the required treatment, the medium was removed and the pericytes were washed with PBS for 2 times. Then, the pericytes were incubated with the mixture of dye (2 μL Calcein-AM and 3 μL PI in 1 mL DMEM) for 15 min at 37°C and the

nuclei were stained with DAPI. Red and green signal indicated the apoptotic cells and alive cells, respectively. The ratio of apoptotic cells was analyzed and relative changes of cell apoptosis among these groups were shown in statistical graph.

**Caspase 3/7 activity assay**

Caspase 3/7 activity was determined using caspase 3/7 activity kit (Caspase-Glo® 3/7 Assay System, G8091, Promega, USA) according to the manufacturer's protocol. Briefly, a total of $1\times10^4$ pericytes/well were planted into the 96-well plates. After the required treatments, 100 μl/well Caspase-Glo 3/7 reagent was added to each well and the plate was put on the plate shaker at room temperature for 1 h. The luminescence signal was measured by a microplate fluorescence reader (FilterMax F5, Molecular Devices, USA).

**Quantitative analysis for cell culture and animal experiments**

For the quantitation of cell culture experiments, the control group was set as 1. The value for experimental group was shown as the relative change compared with the control group. Five visual fields were randomly selected from each group. All experiments were conducted for 4 times. For the quantitation of pericyte ghosts, microaneurysms, and acellular capillaries, we randomly selected 30 different fields from each experimental for the subsequent statistical analysis. The number of acellular capillaries and microaneurysms was expressed per square millimeter of retina and the number of pericyte ghosts was reported per 1000 capillary cells. For the qualification of pericyte coverage, the multiple overlapping images of flat-mounted retinas were captured by a ×10 lens and 5-6 images were arrayed to obtain composite

images of a leaf of retina vessels. Pericyte coverage was calculated by dividing the surface area positive for pericyte marker (NG2) into the surface area positive for endothelial marker (IB4). NG2 signaling was quantified in the vascularized retina from the optic nerve head to distal vascular front at different vascular plexuses. The number of animals per group used in each experimental group was indicated in figure legend. All samples were evaluated in a blinded fashion.

## Supplemental figures

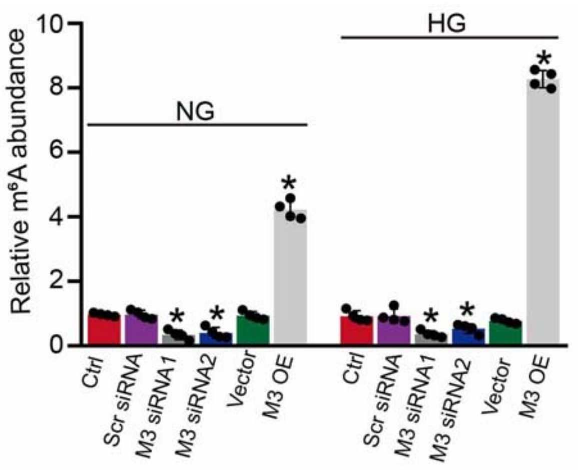


**Figure S1. METTL3 regulated the level of m$^6$A modification in pericytes**

Pericytes were transfected with scrambled (Scr) siRNA, METTL3 siRNA1 (M3 siRNA1), METTL3 siRNA2 (M3 siRNA2), pcDNA 3.1 vector (Vector), pcDNA 3.1-METTL3 (M3 OE), or left untreated (Ctrl), and then incubated with normal culture medium (normal glucose, NG) or 25 mM glucose (high glucose, HG) for 48 h. The levels of m$^6$A RNA modification were determined by the colorimetric quantification (n = 4, *$P$ < 0.05 versus Ctrl group; 1-way ANOVA, Bonferroni test).

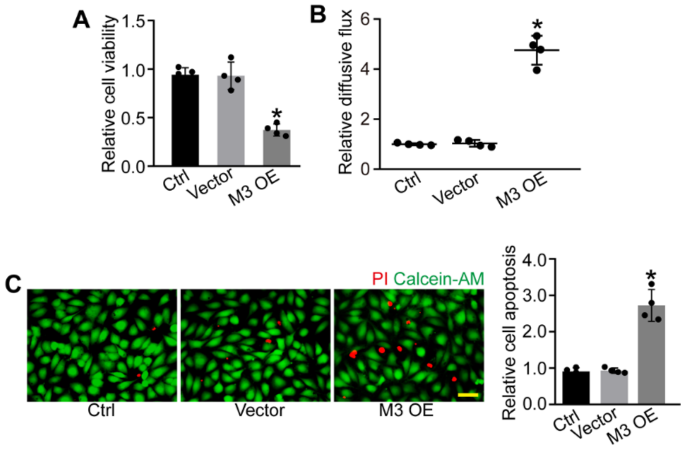


**Figure S2. METTL3 overexpression aggravates high glucose-induced retinal pericyte dysfunction in vitro**

Pericytes were transfected with pcDNA 3.1 vector (Vector), pcDNA 3.1-METTL3 (M3 OE), or left untreated (Ctrl), and then incubated with 25 mM glucose for 48 h. Cell viability was determined by MTT assays (A, n = 4, **P* < 0.05 versus Ctrl group; 1-way ANOVA, Bonferroni test). FITC-Dextran transwell assay was conducted to detect vascular barrier function by the examination of 70-kDa FITC-Dextran leakage (B, n = 4, **P* < 0.05 versus Ctrl group; 1-way ANOVA, Bonferroni test). PI staining was conducted to detect cell apoptosis (C, n = 4, **P* < 0.05 versus Ctrl group; 1-way ANOVA, Bonferroni test).

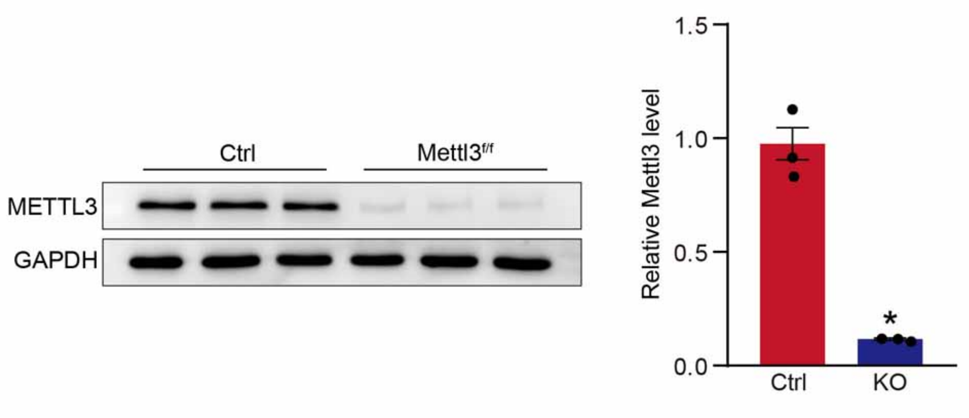


**Figure S3. Detection of *Mettl3* expression in pericytes isolated from the retinas of pericyte-specific *Mettl3* knockout mice**

The retinas were isolated from *Mettl3*$^{+/+}$ mice (Ctrl group) or *Mettl3*$^{f/f}$; *Pdgfrβ*-Cre mice (*Mettl3*$^{f/f}$ group). Western blots were performed to detect the expression of *Mettl3* in the retinas. GAPDH was detected as the control (n = 3 retinas per group, **P* < 0.05 versus Ctrl group). The representative immunoblots were shown.

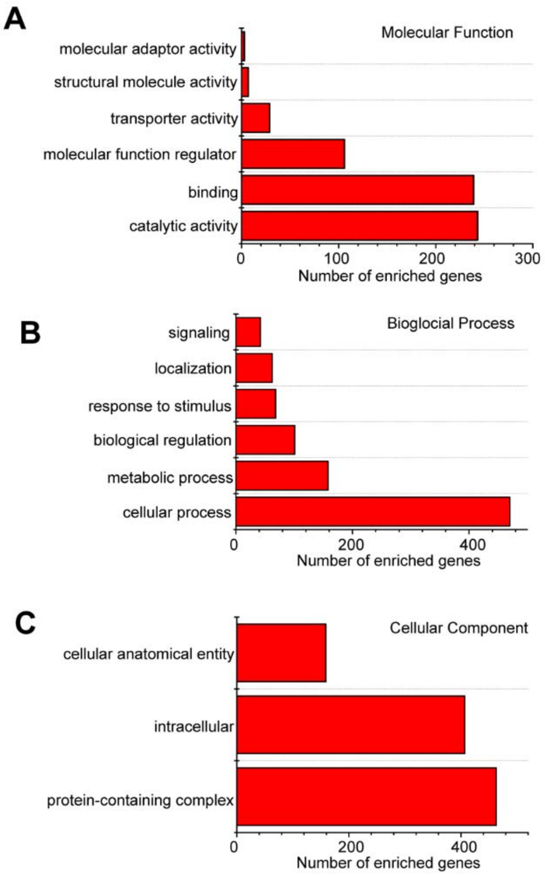


**Figure S4. GO analysis of the differentially expressed genes regulated by METTL3**

(A-C) The functions of the differentially expressed genes regulated by METTL3 were predicted using GO analysis. Gene oncology (GO) enrichment analysis included molecular functions (A), biological processes (B), and cellular components (C).

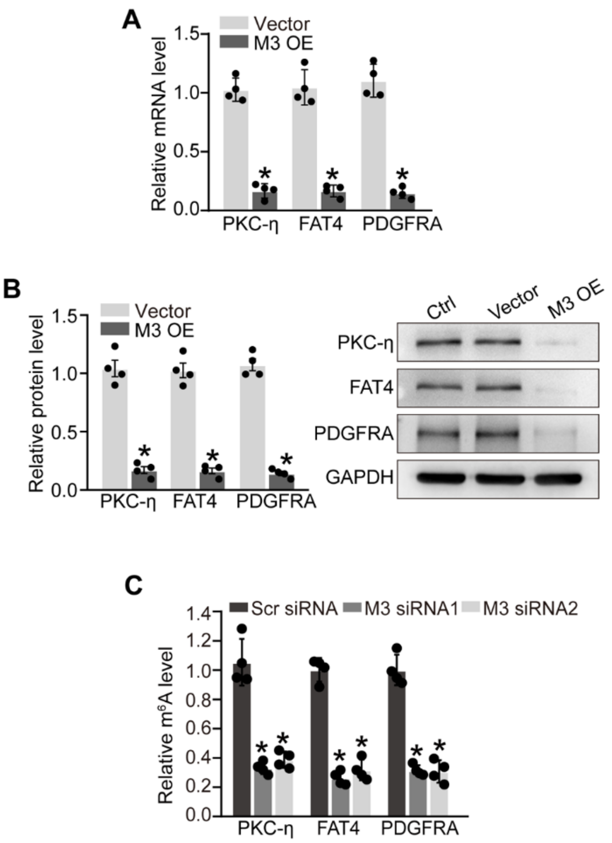


**Figure S5. METTL3 overexpression leads to reduced PKC-η, FAT4, and PDGFRA expression**

(A and B) Pericytes were transfected with pcDNA3.1 (Vector) or pcDNA3.1-METTL3 (M3 OE), or left untreated (Ctrl) for 48 h. qRT-PCR assays (A, n = 4, **P* < 0.05 versus Vector group, Student *t* test*)* and western blots were conduct to detect the levels of PKC-η, FAT4 and PDGFRA. GAPDH was served as internal control (n = 4, **P* < 0.05 versus Ctrl group, 1-way ANOVA, Bonferroni test). (C) MeRIP-qPCR was the $m^6A$ levels of PKC-η, FAT4, and PDGFRA mRNAs in METTL3 silencing pericytes (n = 4, **P* < 0.05 versus Scr group, 1-way ANOVA, Bonferroni test).

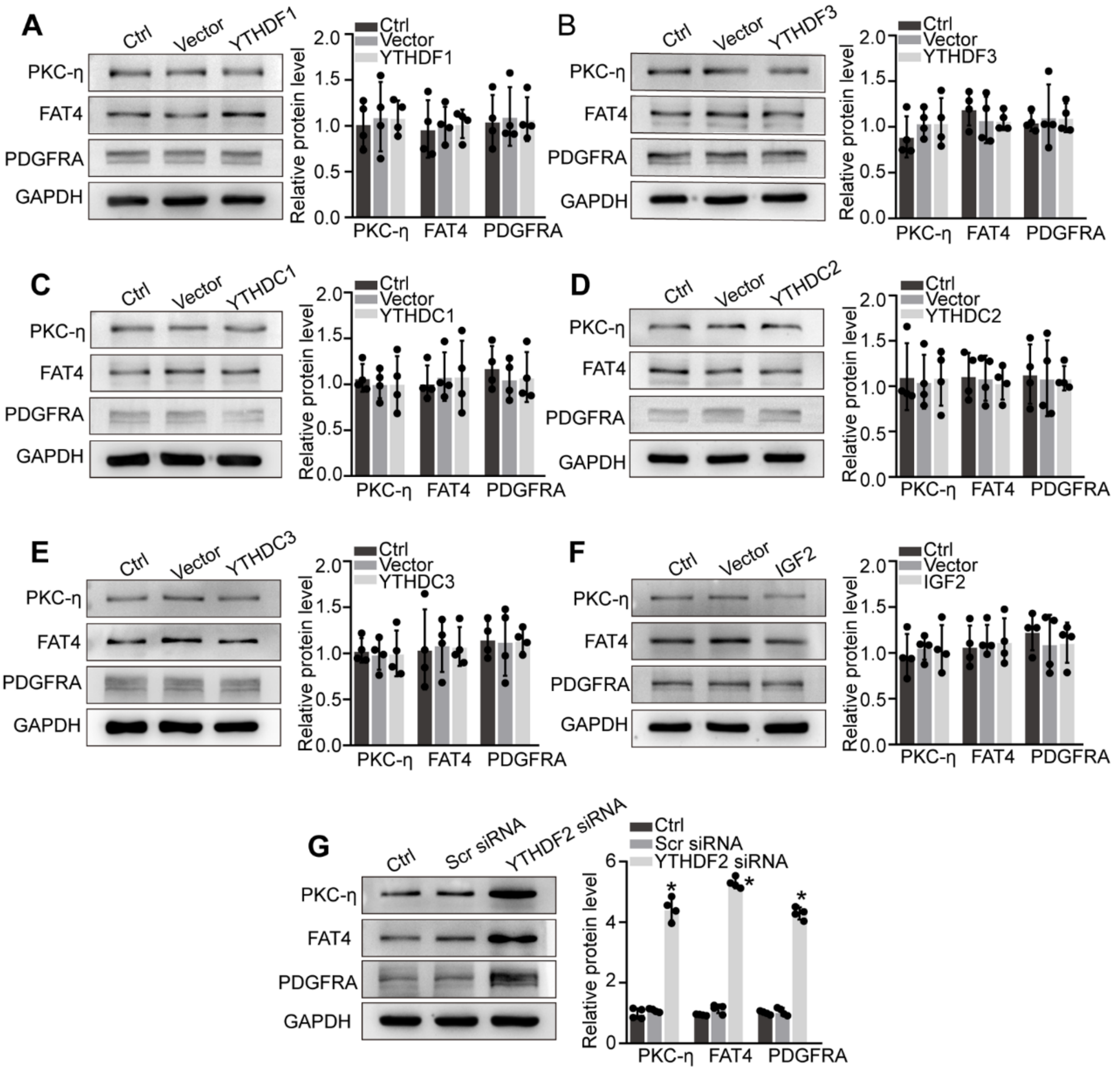


**Figure S6. YTHDF2 but not other reader proteins regulates the expression of PKC-η, FAT4 and PDGFRA**

(A-F) Pericytes were transfected with pcDNA 3.1 vector (Vector), pcDNA 3.1-YTHDF1 (YTHDF1), pcDNA 3.1-YTHDF3 (YTHDF3), pcDNA 3.1-YTHDC1 (YTHDC1), pcDNA 3.1-YTHDC2 (YTHDC2), pcDNA 3.1-YTHDC3 (YTHDF3), pcDNA 3.1-IGF2 (IGF2), or left untreated (Ctrl) for 48 h. The expression levels of PKC-η, FAT4 and PDGFRA were detected by western blots. GAPDH was detected as the internal control. Representative immunoblots and statistical results were shown (n = 4, *$P < 0.05$ versus Ctrl group, 1-way ANOVA, Bonferroni test). (G) Pericytes were transfected with scrambled (Scr) siRNA, YTHDF2 siRNA, or left untreated (Ctrl group) for 48 h. Western blots were conducted to detect the levels of PKC-η, FAT4 and PDGFRA. GAPDH was served as internal control. Representative immunoblots and statistical results were shown (n = 4, *$P < 0.05$ versus Ctrl group, 1-way ANOVA, Bonferroni test).
